# Glycoside Hydrolase Activities in Cell Walls of Sclerenchyma Cells in the Inflorescence Stems of *Arabidopsis thaliana* Visualized *in Situ*

**DOI:** 10.3390/plants3040513

**Published:** 2014-11-12

**Authors:** Alicja Banasiak, Farid M. Ibatullin, Harry Brumer, Ewa J. Mellerowicz

**Affiliations:** 1 Institute of Experimental Biology, University of Wroclaw, 50-328 Wroclaw, Poland; E-Mail: alicja.banasiak@uni.wroc.pl; 2 Division of Glycoscience, School of Biotechnology, Royal Institute of Technology (KTH), AlbaNova University Centre, 106 91 Stockholm, Sweden; E-Mails: ibatullin@omrb.pnpi.spb.ru (F.M.I.); brumer@msl.ubc.ca (H.B.); 3 Biophysics Division, Petersburg Nuclear Physics Institute, National Research Center Kurchatov Institute, Gatchina 188300, Russia; 4 Michael Smith Laboratories, University of British Columbia, 2185 East Mall, Vancouver, BC V6T 1Z1, Canada; 5 Department of Chemistry, University of British Columbia, 2185 East Mall, Vancouver, BC V6T 1Z1, Canada; 6 Department of Forest Genetics and Plant Physiology, Swedish University of Agricultural Sciences, Umea Plant Science Centre, 90183 Umea, Sweden

**Keywords:** cell wall, xylem, wood, sclerenchyma, glycoside hydrolase activity, *in situ* activity

## Abstract

Techniques for *in situ* localization of gene products provide indispensable information for understanding biological function. In the case of enzymes, biological function is directly related to activity, and therefore, knowledge of activity patterns is central to understanding the molecular controls of plant development. We have previously developed a novel type of fluorogenic substrate for revealing glycoside hydrolase activity *in planta*, based on resorufin β-glycosides Here, we explore a wider range of such substrates to visualize glycoside hydrolase activities in *Arabidopsis* inflorescence stems in real time, especially highlighting distinct distribution patterns of these activities in the secondary cell walls of sclerenchyma cells. The results demonstrate that β-1,4-glucosidase, β-1,4-glucanase and β-1,4-galactosidase activities accompany secondary wall deposition. In contrast, xyloglucanase activity follows a different pattern, with the highest signal observed in mature cells, concentrated in the middle lamella. These data further the understanding of the process of cell wall deposition and function in sclerenchymatic tissues of plants.

## 1. Introduction

Knowledge of the precise localization of specific enzymatic activities in distinct cell types and cell compartments of plants is a prerequisite for understanding the biological functions of these enzymes and their encoding genes [1]. Plant genomes are renowned for encoding an abundance of glycoside hydrolases and transglycosidases, which probably reflects the need for cell wall remodeling [2,3,4,5,6,7]. Most work on cell wall remodeling by these enzymes has focused on changes during primary cell wall expansion [8,9,10,11,12] and fruit ripening [13]. However, there is an increasing awareness of the importance of secondary cell wall remodeling for plant development by enzymes, such as xyloglucan *endo*-transglycosylase (XET) [14,15,16] and xylan *endo*-transglycosylase [17]. For example, xyloglucan *endo*-transglycosylase activity in gelatinous fibers has been found to play an essential role in the tension wood function and was proposed to be involved in the generation of contractile forces by gelatinous fibers [15,18,19,20].

The abundance of different classes of glycoside hydrolases in secondary cell walls has become evident from proteomic analyses [21,22]. Here, we present *in situ* activity tests using a range of fluorogenic substrates to determine the tissue and cell distribution of major classes of cell wall-active enzymes in mature inflorescence stems of *Arabidopsis*, including β-1,4-glucosidases, β-1,4-glucanases, β-1,4-galactosidases and xyloglucanases. Most of these activities were associated with cell wall deposition in sclerenchyma cells, but were notably also found in the walls of mature cells. These results indicate that sclerenchyma cell wall remodeling is tightly associated with cell wall biosynthesis and that it may continue *post mortem*.

## 2. Results

### 2.1. Biosynthesis of Different Fluorogenic Substrates

**Figure 1:**
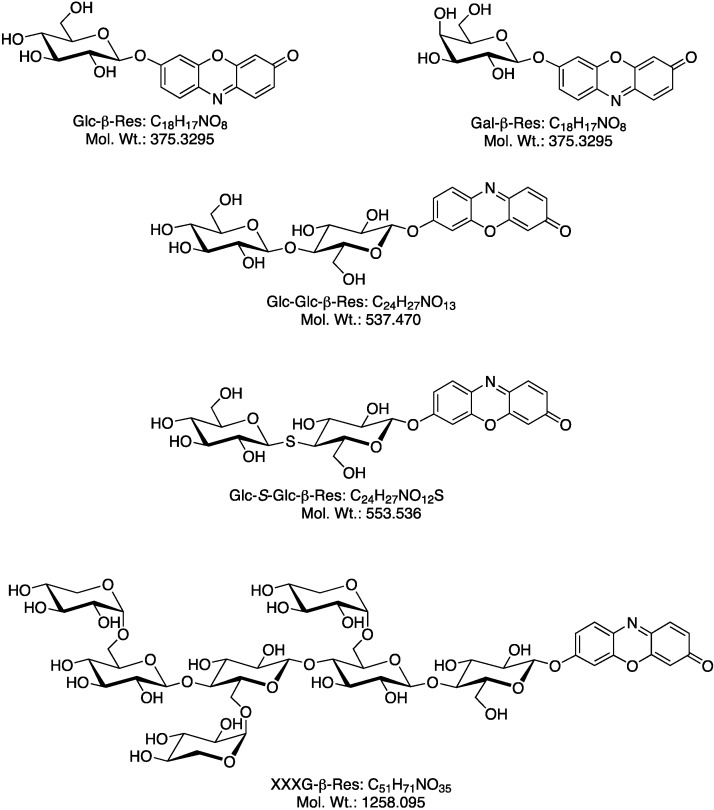
Structures of the fluorogenic substrates used in this study (Glc*-*β-Res; Glc-Glc-β-Res; Glc-*S*-Glc-β-Res; Gal-β-Res; XXXG-β-Res).

A set of resorufin β-glycosides (Figure 1), serving as substrates for different glycoside hydrolases, was synthesized according to previously published protocols [1]. The release of the resorufin aglycone (p*K*_a_ 5.8) by glycoside hydrolase activity results in a significant increase in fluorescence due to the resorufin anion (extinction: 62,000 m^−1^·cm^−1^; excitation maximum: 571 nm; emission maximum: 585 nm; quantum yield: 0.74). Five different resorufin glycosides were synthesized: (1) glucose-β-resorufin (Glc*-*β-Res, substrate for β-1,4-glucosidases); (2) cellobiose-β-resorufin (Glc-Glc-β-Res, substrate for β-1,4-glucanases [23], or β-1,4-glucosidases, via sequential cleavage); (3) thio-cellobiosyl-β-resorufin (Glc-*S*-Glc-β-Res, substrate for β-1,4-glucanases with a non-hydrolyzable internal glycosidic bond); (4) galactose-β-resorufin (Gal-β-Res, substrate for β-1,4-galactosidases); and (5) XXXG-β-resorufin (XXXG-β-Res, heptasaccharide-containing substrate for xyloglucan *endo*-hydrolases [1]; see Tuomivaara *et al.* [24] for xyloglucan oligosaccharide nomenclature).

### 2.2. Optimization of in Situ Reaction Conditions for the Different Fluorogenic Substrates

Measurements of enzyme activity in real time were carried out on cross-sections through the basal part of *Arabidopsis thaliana* inflorescence stems. Previous *in vitro* studies using resorufin glycosides have established conditions when the fluorescence signal due to resorufin release depended linearly on time and enzyme concentration [1]. For the semi-quantitative analysis using confocal microscopy performed here, it was also necessary to determine the parameters under *in situ* conditions to ensure that signal saturation was avoided.

The optimal incubation time of tissue with the substrate needed for the enzymatic release of resorufin was first established based on the observation of time series. For this purpose, the substrates were dissolved in 25 mM MES buffer at pH 6.5 to a final concentration of 4.7 × 10^−4^ M and incubated with fresh hand sections of inflorescence stems for 1.5 h with periodic scanning by the confocal microscope. The signal from each substrate first increased with time, then reached a plateau or slightly decreased, possibly due to diffusion of the resorufin anion, or self-quenching or bleaching of the fluorophore. The time after which the fluorescence intensity was the strongest was different for each substrate, but the substrates could be split into fast and slow groups. The fast group included Glc-β-Res, Glc-Glc-β-Res and Gal-β-Res, which gave clear signals already after 2 min, with signal saturation occurring after about 20 min. The slow group included Glc-*S*-Glc-β-Res and XXXG-β-Res, which required approximately 10 min to observe the first apparent signals, with the maximum signal typically arising after 60–90 min. These differences in the reaction times reflect differences in the abundance of the different enzymatic activities in inflorescence stem tissues. Thus, reaction times of either 20 or 60 min were chosen, one factor in the optimized reaction conditions for the fast- and slow-consumed substrates, respectively (Table 1).

**Table 1:** Optimal reaction conditions (reaction times, substrate concentrations) for the signal strength and dependence of the signal strength on pH for the different glycoside resorufin substrates in *Arabidopsis* stem sections.

Reaction Times	Substrates	Concentration (M)	pH				
			5.0	5.5	6.0	6.5	7.0
20 min	Glc-β-Res	9.3 × 10^−5^	++	++	+++	+++	++
	Glc-Glc-β-Res	9.3 × 10^−5^	++	++	+++	+++	++
	Gal-β-Res	4.7 × 10^−5^	++	+++	+++	++++	++
60 min	Glc-*S*-Glc-β-Res	9.3 × 10^−4^	-	-	+	+	++
	XXXG-β-Res	9.3 × 10^−4^	-	-	+	++	+

Free resorufin has a pKₐ value of 5.8, and the observable fluorescence is due to the resorufin anion (presenting a 50% abundance at pH 5.8), while many glycoside hydrolases exhibit acidic pH-rate optima. Therefore, five different pH values, between pH 5.0 and pH 7.0, were tested for each substrate (Table 1). The highest signal was typically observed when the pH was slightly acidic (6.0–6.5), likely reflecting a compromise between resorufin ionization and enzyme activity, with the exception of Glc-*S*-Glc-β-Res, which had the strongest signal at pH 7.0.

A third factor that is important for the precise location of a particular enzymatic activity is the substrate concentration. Four different concentrations were tested for each of the substrates at optimal pH with the time series: 4.7 × 10^−5^ M, 9.3 × 10^−5^ M, 4.7 × 10^−4^ M and 9.3 × 10^−4^ M, and the “signal-to noise” was visually evaluated. The lowest concentrations of acceptable signals are listed in Table 1. Substrates with long reaction times required the highest concentrations, likely to ensure enzyme saturation. In the case of Gal-β-Res, the fluorescence was very intense, and the diffusion of released resorufin was observed; therefore, the lowest of the concentrations tested, 4.7 × 10^−5^ M, was indicated for this substrate.

### 2.3. In Situ Enzymatic Activity Distribution Observed in Real Time at the Tissue Level in Inflorescence Stems of Arabidopsis thaliana

**Figure 2:**
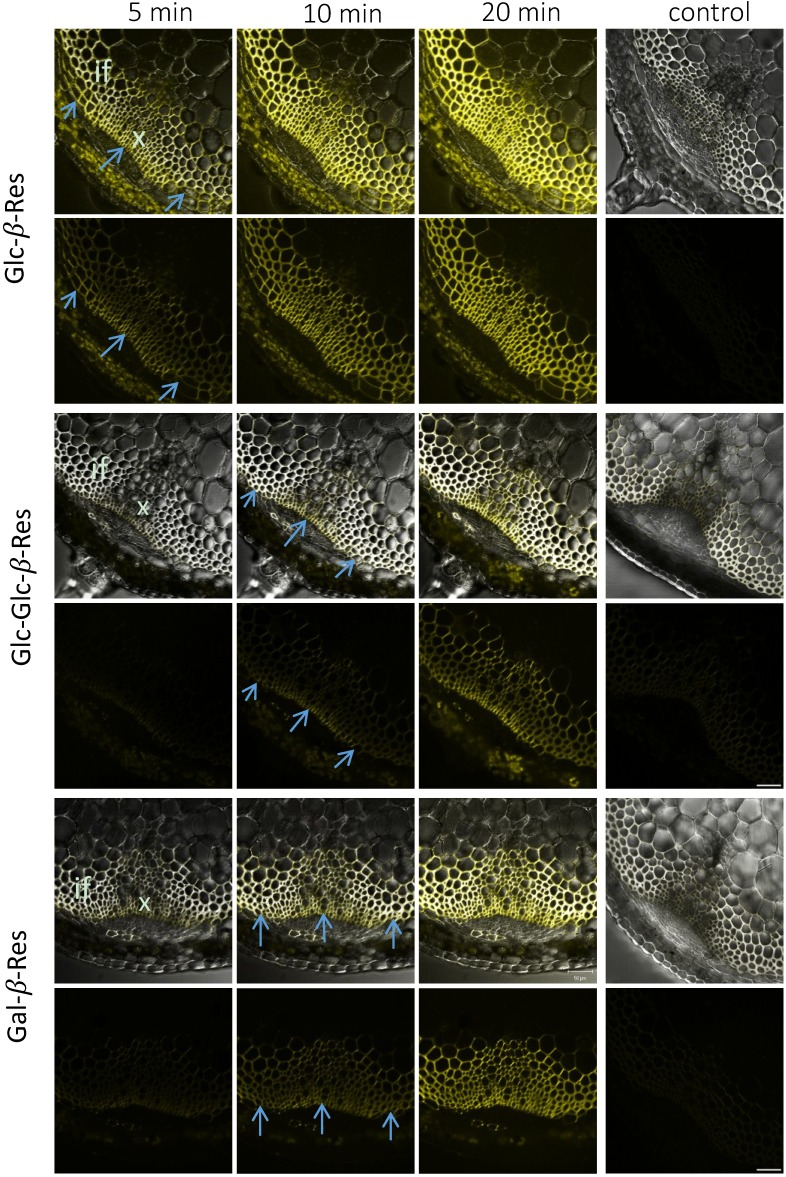
Distribution of signals in the inflorescence stem sections of *Arabidopsis* from Glc-β*-*Res, Glc-Glc*-*β*-*Res and Gal-β*-*Res substrates after incubations for the specified durations. The control sections were boiled for 30 min before incubation with the specified substrate for 20 min. Upper rows represent the transmitted light channel and the superimposed yellow fluorescence channel corresponding to resorufin signals, and the lower rows represent the yellow channel only. Blue arrows show differentiating sclerenchyma cells. if, interfascicular fibers; x, xylem. Bar = 50 μm.

**Figure 3:**
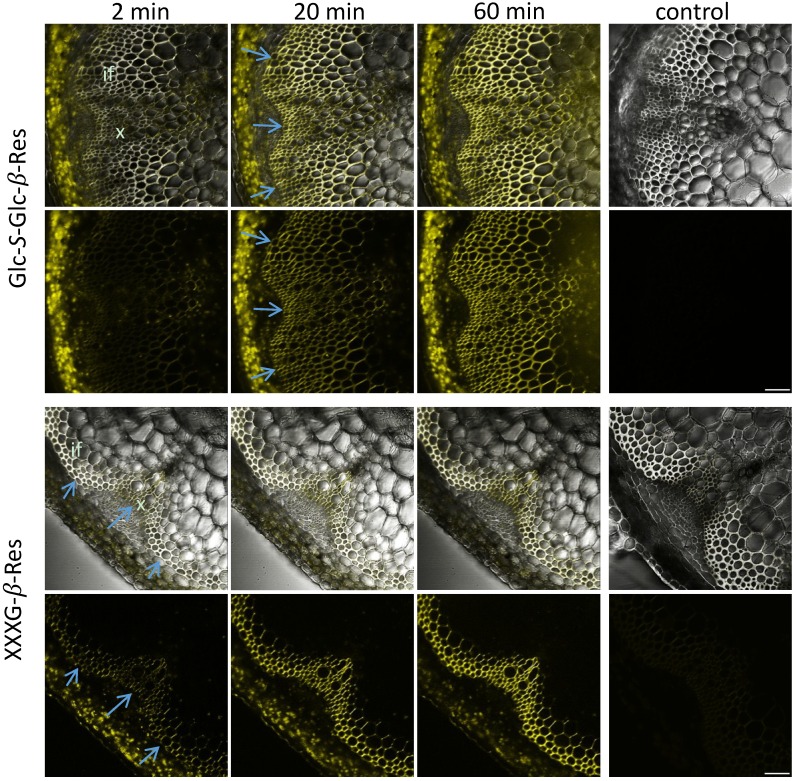
Distribution of signals in the inflorescence stem sections of *Arabidopsis* from Glc-*S*-Glc-β-Res and XXXG-β-Res substrates after incubations for the specified durations. The controls were boiled for 30 min before incubation for 60 min. Upper rows represent the transmitted light channel and the superimposed yellow fluorescence channel corresponding to resorufin signals, and the lower rows represent the yellow channel only. Blue arrows show differentiating sclerenchyma cells. if, interfascicular fibers; x, xylem. Bar = 50 μm.

The fluorescence signal reflecting glycoside hydrolase activity using all five substrates was detected primarily in the cell walls of interfascicular fibers and xylem cells, *i.e.*, in a cylinder of sclerenchyma tissues (Figure 2 and Figure 3). Some intracellular signals, associated with plastids, were also observed in the cortical tissues. However, this signal was observed without any substrate (Supplemental Material 1), and all of these signals were absent in heat-treated sections (Figure 2 and Figure 3), which suggests that they were dependent on heat-labile compounds in plant tissues. Unlike the cell wall-localized signals, the intracellular signals in the cortex did not display the time-dependent increase expected for enzymatically-released resorufin. We therefore interpret these intracellular signals as resulting from autofluorescence of heat-labile pigments and discounted them from further analysis.

The signal evolution with Glc-β*-*Res, Glc-Glc*-*β*-*Res, Glc-*S*-Glc*-*β*-*Res and Gal-β*-*Res in stem sections was similar. Signals were first detected in the outer cylinder of sclerenchyma tissues, corresponding to the youngest cells developing a secondary cell wall (blue arrows in Figure 2 and Figure 3). With ongoing incubation, the signal became more intense and gradually appeared throughout all of the cells of the sclerenchyma. This labeling pattern was distinct from that observed with XXXG*-*β*-*Res, which produced a signal only in the mature sclerenchyma cells and had an unchanged distribution pattern throughout the whole incubation time (Figure 3). The difference in signal resulting from XXXG*-*β*-*Res and the other substrates can be also distinctly seen in time-lapse movies (Supplemental Materials 2–6).

### 2.4. In Situ Enzymatic Activity Observed in Real Time on the Cell Level in Interfascicular Fibers of Inflorescence Stems in Arabidopsis thaliana

**Figure 4:**
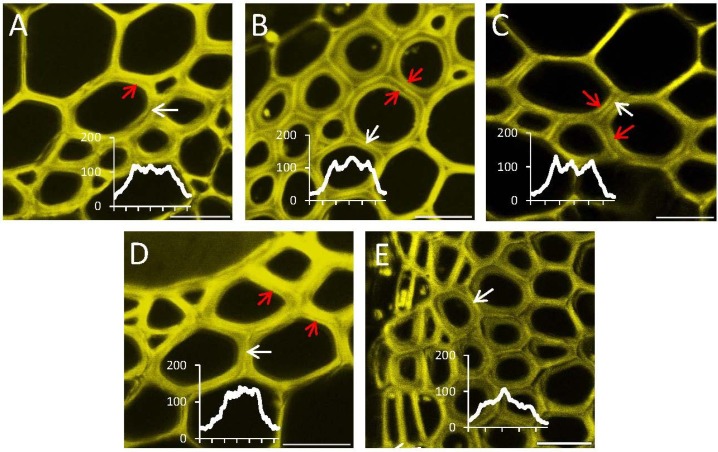
Distribution of signals from different glycoside hydrolase activities in the cell walls of interfascicular fibers in *Arabidopsis* stems. (**A**) Glc-β-Res; (**B**) Glc-Glc-β-Res; (**C**) Glc-*S*-Glc-β-Res; (**D**) Gal-β-Res; (**E**) XXXG-β-Res. All reactions were carried out in optimal conditions (Table 1). The yellow fluorescence channel with resorufin signals is shown. Signals from the compound middle lamella are shown with the white arrows, and signals from the recently formed secondary wall layers are shown with red arrows. Intensity profiles through the cell wall area, shown by the white arrows, are included to visualize the labeling patterns in the cell wall better. Bar = 50 μm.

The distribution of signals reflecting the different glycoside hydrolytic activities was additionally analyzed at the cellular level in the interfascicular fibers of the *Arabidopsis* inflorescence stems*.* For all of the substrates, activity was observed in several cell wall layers (Figure 4), with prominent signals observed in the compound middle lamella (white arrows) and in the innermost, freshly deposited cell wall layer (red arrows). The relative intensity between these two positions varied among the substrates. Glc-Glc*-*β*-*Res and Glc-*S*-Glc-β-Res gave the strongest signals in the innermost layer of secondary wall, whereas the signal from XXXG-β-Res was most prominent in the compound middle lamella. The signals after Gal-β*-*Res and Glc-β*-*Res application were intermediate between these two divergent patterns. These results highlight that resorufin*-*β*-*glycosides, differing in their glycon component, can be used to identify individual glycoside hydrolase activities in real time, not only at the tissue level, but also at the cellular level.

## 3. Discussion

The *in situ* distribution patterns of different glycoside hydrolase activities detected with the fluorogenic substrates in the sclerenchyma of *Arabidopsis* provide novel information about cell wall biochemistry in this tissue. Localized signals observed in cell walls most likely result from the activities of enzymes that are not free to diffuse. These enzymes could be either ionically bound to cell walls, as is known for some cellulases, pectate lyases, pectin methyl esterases or alpha-expansins, or be covalently-bound to the substrate, as is known for XET, or else be anchored in the plasma membrane by a transmembrane domain, like KOR1, or by a GPI anchor, like PMR6 [4,8].

In particular, the results indicate that developing secondary walls have an abundance of β-1,4-glucosidase activities (Figure 3 and Figure 4; Supplemental Material 2). β-glucosidase activities were proposed to be present in growing primary walls [25] and have been extracted in the proteome of mature stems [21], but to our knowledge, this is the first report of the specific localization of these activities in the compound middle lamella and in developing secondary wall layers in the sclerenchyma cells. These observations imply that cell wall polysaccharide fragments released by various *endo*-glucanases or other glucosidases undergo trimming to release glucose to the apoplast, possibly followed by uptake and recycling.

The importance of β-1,4-galactosidase activity has been documented during cell wall formation in flax fibers [26,27]. These fibers represent a gelatinous fiber type (G-fibers), as opposed to the S-fibers of sclerenchyma composed of secondary walls rich in xylan [20]. β-1,4-galactosidase activity has been shown to be essential for the normal development of G-fibers, for cellulose crystallinity in the gelatinous layer and for fiber mechanical strength [26,27]. Our present data, revealing this activity in the sclerenchyma cells in *Arabidopsis* (Figure 2 and Figure 4, Supplemental Material 5), imply a role for β-1,4-galactosidase also in S-fibers with xylan-type secondary walls. We observed galactosidase activity mainly in the compound middle lamella, where the majority of β-1,4-galactan is known to be located in S-fibers [20]. However, some signals were also detected in the innermost secondary wall layers. These signals may correspond to galactosidases being currently produced and delivered to cell walls for the modification of compound middle lamella.

Glc-Glc-β-Res may act as a substrate for both β-1,4-glucosidases and β-1,4-glucanases, whereas the non-cleavable internal thioglycosidic bond of Glc-*S*-Glc-β-Res confers activity specific to β-1,4-glucanases. The comparison of the signal distribution across Glc-β-Res, Glc-Glc-β-Res and Glc-*S*-Glc-β-Res (Figure 2, Figure 3 and Figure 4; Supplemental Materials 2–4) allows us to conclude that both β-1,4-glucosidases and β-1,4-glucanases are active during secondary wall development in sclerenchyma cells, but that β-1,4-glucanases are less abundant or less active, at least against these artificial fluorogenic substrates. Interestingly, the β-1,4-glucanase-specific signal was highest in the innermost secondary cell wall layers for all activities tested (Figure 4). It is tempting to speculate that one of these glucanases may be KOR1, a membrane-bound cellulase implicated in cellulose biosynthesis in primary and secondary walls [28]. In secondary walls, KOR1 was found to reduce cellulose crystallinity, although the mechanisms are still not fully understood [28,29].

The most intriguing finding was the lack of xyloglucanase activity during sclerenchyma cell development at the primary-wall stage, when xyloglucan deposition is highest, while this activity was highest in mature sclerenchyma cells (Figure 3 and Figure 4, Supplementary Material 6). We previously observed an absence of xyloglucanase activity in developing fibers [1]. Here, we have confirmed that finding and further observed that xyloglucanase activity was concentrated in the middle lamella of mature fibers (Figure 3 and Figure 4; Supplemental Material 6). Currently, the only identified xyloglucanases are encoded by members of Group IIIa of the *XTH* gene family, a subgroup of glycoside hydrolase family 16 [6,30]. In some species with xyloglucan-rich seeds, such as nasturtium, xyloglucanase activity is detected in the storage cell walls during seed germination, when the polysaccharide reserve is mobilized [1]. In nasturtium, the corresponding Group IIIa enzymes, *Tm*NXG1 and *Tm*NXG2, have been biochemically and structurally characterized [30]. *Arabidopsis* has only two Group IIIa members, *XTH31* and *XTH32,* and *XTH31* is the main gene expressed in elongating roots [31]. *Arabidopsis* also lacks a mixed-function *endo*-xyloglucanase, ancestral to the *XTH* gene family in GH16, that is found in other dicots [32]. Phenotypic analysis of a *XTH31/XTH32* double mutant failed to identify a precise role of xyloglucanase activity in the growing roots [31]. Recently, the *XTH31* gene product was implicated in Al^3+^ resistance by interaction with the xyloglucan *endo*-transglycosylase XTH17 and modulation of the xyloglucan content of root cell walls [33,34]. It is possible that modulation of xyloglucan content in mature sclerenchyma may also occur. In support of this hypothesis, a decrease in xyloglucan was observed in mature xylem cells of aspen when *Ptt*XET34 was ectopically overexpressed [16]. Elucidation of the physiological role of such xyloglucan modulation is an exciting research topic for future studies.

## 4. Experimental Section

### 4.1. Substrates Synthesis

Resorufinyl glycosides were prepared from corresponding per-*O*-acetylated glycosyl bromides using glycosylation under phase-transfer catalysis and subsequent deacetylation, as described previously [1]. NMR and MS data of synthesized compounds were in full agreement with previously published data [1,23,35]. Hepta-*O*-acetyl-4-thio-4-*S*-cellobiosyl bromide for Glc-*S*-Glc-β-Res synthesis was prepared from methyl 2,3,6-tri-*O*-benzoyl-4-*S*-(2,3,4,6-tetra-*O*-acetyl-β-d-glucopyranosyl)-4-thio-β-d-glucopyranoside according to Orgeret *et al.* [36]. Novel NMR data for resorufinyl 2,3,6-tri-*O*-acetyl-4-*S*-(2,3,4,6-tetra-*O*-acetyl-β-d-glucopyranosyl)-4-thio-β-d-glucopyranoside and resorufinyl 4-*S*-(β-d-glucopyranosyl)-4-thio-β-d-glucopyranoside (Glc-*S*-Glc-β-Res) are given in Supplementary Material 7. NMR and MS data of synthesized compounds were in full agreement with previously published data [1,23,35].

### 4.2. Material and Growth Conditions

Seeds of *Arabidopsis* after stratification at 4 °C for 3 days in 0.1% agar were sown in pots filled with soil and placed in a room with long-day conditions (16 h day/8 h night) and a photon flux density of 120 to 150 µE m^−2^·s^−1^. Plants were grown in 80% relative humidity and a constant temperature at 22 °C, for 6–7 weeks until Growth Stage 6.3 [37].

### 4.3. Optimization of the Enzymatic Reaction Conditions

Fresh hand cross-sections from the basal part of the inflorescence stems of *Arabidopsis* were placed on slides with 50 µL of the substrate, covered with a cover slip, the edges of which were sealed with nail polish. The fluorescent product of enzymatic activity (resorufin) was detected by confocal laser scanning microscopy (LSM-510, Carl Zeiss, Germany) at 567 nm excitation and a 580-nm detection wavelength. A transmitted light channel was used simultaneously to visualize the anatomical details. Experiments were repeated at least three times for each substrate, using low (10× objective lens ×8) and high (40× objective lens ×8) magnification. The profiles were made using ImageJ. Control sections were boiled 30 min at 95 °C in 25 mM MES buffer before incubation in the substrate solutions. Autofluorescence control sections were scanned without incubation in substrates with the same scanning confocal microscope settings as the experimental sections.

#### 4.3.1. The Reaction Time Optimization

All tested substrates were dissolved in 25 mM 2-morpholino-ethanesulfonic acid (MES) buffer at pH 6.5 and a 4.7 × 10^−4^ M concentration. Sections immersed in substrates were scanned at five minute intervals, using the “time series” option.

#### 4.3.2. pH Optimization

Substrates were dissolved in 25 mM MES at a 4.7 × 10^−4^ M concentration at different pH levels: 5.0, 5.5, 6.0, 6.5, 7.0. The incubation time for Glc-β-Res, Glc-Glc-β-Res and Gal-β-Res was 20 min, and for Glc-*S*-Glc-β-Res and XXXG-β-Res, it was 60 min.

#### 4.3.3. Substrate Concentration Optimization

Each studied substrate was dissolved at the optimal pH to a final concentration of 9.3 × 10^−4^ M, 4.7 × 10^−4^ M, 9.7 × 10^−5^ M and 4.7 × 10^−5^ M with 25 mM MES buffer. Sections were incubated for 20 min for Glc-β-Res, Glc-Glc-β-Res and Gal-β-Res or 60 min for Glc-*S*-Glc-β-Res and XXXG-β-Res, chosen based on the observations of the time series.

## 5. Conclusions

In this work, we have demonstrated the presence of several glycoside hydrolases in sclerenchyma cell walls in *Arabidopsis* by direct, real-time observation of their activities using a library of fluorogenic probes. The results support the notion that cell walls are being constantly remodeled, not only during cell wall deposition, but also when cells are thought to be fully “mature.” The physiological role of the remodeling is still not fully understood for all polysaccharides. It could involve the release of mechanical stress accrued during growth, recycling of carbohydrates and, relatedly, modulation of oligosaccharin signaling, or regulation of cell wall ionic content. In sclerenchyma cells, these modifications might also impact the lignification process.

The results described here demonstrate that the resorufin glycosides are effective for the identification of diverse hydrolytic activities at cellular resolution. Broader application of these substrates will facilitate a better understanding of the post-synthetic modification of the cell wall under different situations, including normal morphogenesis and in response to changing environmental conditions, e.g., pathogen attack and abiotic stress.

## Supplementary Files

Supplementary File 1
