# Supplementary material for: Glycoside Hydrolase Activities in Cell Walls of Sclerenchyma Cells in the Inflorescence Stems of Arabidopsis thaliana Visualized in Situ
Source: Plants (Basel). 2014 Nov 12;3(4):513–25. doi: 10.3390/plants3040513 (PMC4844284; doi:10.3390/plants3040513)
Supplement: Supplementary File 1 [file plants-03-00513-s001.zip › plants-66741-supplementary-layout/Supplementary Material-1.pdf]

Supplemental Material 1. Autofluorescence of cortical cells in Arabidopsis stem sections. Micrographs show the transmitted light channel (A) and fluorescence seen in the resorufin channel (B).

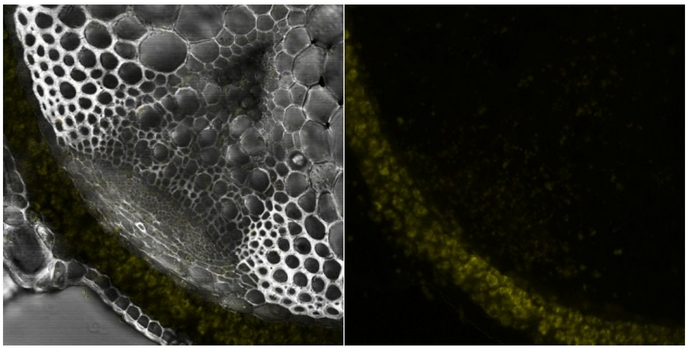

A B

© 2014 by the authors; licensee MDPI, Basel, Switzerland. This article is an open access article distributed under the terms and conditions of the Creative Commons Attribution license (http://creativecommons.org/licenses/by/4.0/).
