# Supplementary material for: Glycoside Hydrolase Activities in Cell Walls of Sclerenchyma Cells in the Inflorescence Stems of Arabidopsis thaliana Visualized in Situ
Source: Plants (Basel). 2014 Nov 12;3(4):513–25. doi: 10.3390/plants3040513 (PMC4844284; doi:10.3390/plants3040513)
Supplement: Supplementary File 1 [file plants-03-00513-s001.zip › plants-66741-supplementary-layout/Supplementary Material-7.pdf]

Supplementary Material 7. NMR signals for Resorufinyl 2,3,6-tri-*O*-acetyl-4-*S*-(2,3,4,6-tetra-*O*-acetyl-*β*-D-glucopyranosyl)-4-thio-*β*-D-glucopyranoside and Resorufinyl 4-*S*-(*β*-D-glucopyranosyl)-4-thio-*β*-D-glucopyranoside (Glc-*S*-Glc-*β*-Res).

Resorufinyl 2,3,6-tri-O-acetyl-4-S-(2,3,4,6-tetra-O-acetyl-β-D-glucopyranosyl)-4-thio-β-D-glucopyranoside. ^{1}H-NMR (CDCl3, 500 MHz): δ (ppm) 7.64 (d, 1H, J = 9.1 Hz), 7.35 (d, 1H, J = 9.8 Hz), 6.93 (m, 2H), 6.92 (d, 1H, J = 2.5 Hz), 6.78 (dd, 1H, J_1 = 9.8 Hz, J_2 = 2.0 Hz), 6.22 (d, 1H) (6H of resorufin), 5.21 (dd, 1H, $J_{3,4}$ = 10.6 Hz, H-3), 5.17 (dd, 1H, $J_{2,3}$ = 8.8 Hz, H-2), 5.16 (d, 1H, $J_{3',4'}$ = 9.7 Hz, H-3'), 5.12 (d, 1H, $J_{1,2}$ = 7.7 Hz, H-1), 4.99 (t, 1H, $J_{4',5'}$= 9.9 Hz, H-4'), 4.88 (dd, 1H, $J_{2',3'}$ = 9.1 Hz, H-2'), 4.71 (d, 1H, $J_{1',2'}$ = 10.0 Hz, H-1'), 4.64 (dd, 1H, $J_{6a,6b}$ = 12.1 Hz, H-6a), 4.31 (dd, 1H, H-6b), 4.23 (dd, 1H, $J_{6'a,6'b}$ = 12.4 Hz, H-6'a), 4.08 (dd, 1H, H-6'b), 4.02 (ddd, 1H, $J_{5,6a}$ = 2.1 Hz, $J_{5,6b}$ = 6.0 Hz, H-5), 3.70 (ddd, 1H, $J_{5',6'a}$ = 2.4 Hz, $J_{5',6'b}$ = 5.1 Hz, H-5'), 2.97 (dd, 1H, $J_{4,5}$ = 10.7 Hz, H-4), 2.05, 2.04, 2.03, 2.01, 1.98, 1.96, 1.93 (7 × CH_3). ^{13}C-NMR ($CDCl_3$, 125 MHz): δ (ppm) 170.39, 170.20, 170.04, 170.01, 169.36, 169.33, 169.19 (7 × CO-Ac), 186.22, 159.92, 149.52, 146.89, 145.11, 134.81, 134.70, 131.58, 129.58, 115.10, 106.93, 103.55 (12 × C-Ar), 98.31 (C-1), 81.71 (C-1′), 75.93, 74.67, 73.59, 72.36, 70.34, 70.04, 68.14 (C-2, C-2′, C-3, C-3′, C-4′, C-5, C-5′), 63.46, 62.12 (C-6, C-6′), 45.91 (C-4), 21.45, 20.73, 20.72, 20.66, 20.59, 20.55, 20.54, 20.49 (7 × CH_3-Ac).ESI Q-TOF HRMS (m/z): 870,1907 (870,1891 calculated for $C_{38}H_{41}NNaO_{19}S$ $[M+Na]^+$).

Resorufinyl 4-S-(β-D-glucopyranosyl)-4-thio-β-D-glucopyranoside (Glc-S-Glc-β-Res). ^{13}C-NMR (DMSO-d_6, 125 MHz): δ (ppm) 185.36, 160.82, 149.64, 145.75, 144.90, 134.92, 133.86, 131.21, 128.45, 114.67, 105.71, 102.50 (12C of resorufin), 99.35 (C-1), 83.22(C-1′), 80.58, 77.52, 76.81, 74.02, 73.30, 72.51, 69.78(C-2, C-2′, C-3, C-3′, C-4′, C-5, C-5′), 61.04, 60.79 (C-6, C-6′), 45.24 (C-4). ESI Q-TOF HRMS (m/z): 576,1132 (576,1152 calculated for $C_{24}H_{27}NNaO_{12}S$ $[M+Na]^+$).

© 2014 by the authors; licensee MDPI, Basel, Switzerland. This article is an open access article distributed under the terms and conditions of the Creative Commons Attribution license (http://creativecommons.org/licenses/by/4.0/).
