# Supplementary material for: Glycoside Hydrolase Activities in Cell Walls of Sclerenchyma Cells in the Inflorescence Stems of Arabidopsis thaliana Visualized in Situ
Source: Plants (Basel). 2014 Nov 12;3(4):513–25. doi: 10.3390/plants3040513 (PMC4844284; doi:10.3390/plants3040513)
Supplement: Supplementary File 1 [file plants-03-00513-s001.zip › plants-66741-supplementary-layout/Supplementary Material.pdf]

Supplementary Materials

1. Autofluorescence of cortical cells in *Arabidopsis* stem sections.
2. Time series *in situ* *β*-1,4-glucosidase activity (Glc-*β*-Res) in the inflorescence stem of *Arabidopsis*
3. Time series *in situ* *β*-1,4-glucosidase and *β*-1,4-glucanase activities (Glc-Glc-*β*-Res) in the inflorescence stem of *Arabidopsis*
4. Time series *in situ* *β*-1,4-glucanase activity (Glc-*S*-Glc-*β*-Res) in the inflorescence stem of *Arabidopsis*
5. Time series *in situ* *β*-1,4-galactosidase activity (Gal-*β*-Res) in the inflorescence stem of *Arabidopsis*
6. Time series *in situ* xyloglucanase activity (XXXG-*β*-Res) in the inflorescence stem of *Arabidopsis*
7. NMR data for resorufinyl 2,3,6-tri-*O*-acetyl-4-*S*-(2,3,4,6-tetra-*O*-acetyl-*β*-D-glucopyranosyl)-4-thio-*β*-D-glucopyranoside and resorufinyl 4-*S*-(*β*-D-glucopyranosyl)-4-thio-*β*-D-glucopyranoside (Glc-*S*-Glc-*β*-Res).

© 2014 by the authors; licensee MDPI, Basel, Switzerland. This article is an open access article distributed under the terms and conditions of the Creative Commons Attribution license (http://creativecommons.org/licenses/by/4.0/).
