# Supplementary figures and images for: Glycoside Hydrolase Activities in Cell Walls of Sclerenchyma Cells in the Inflorescence Stems of Arabidopsis thaliana Visualized in Situ
Source: Plants (Basel). 2014 Nov 12;3(4):513–25. doi: 10.3390/plants3040513 (PMC4844284; doi:10.3390/plants3040513)

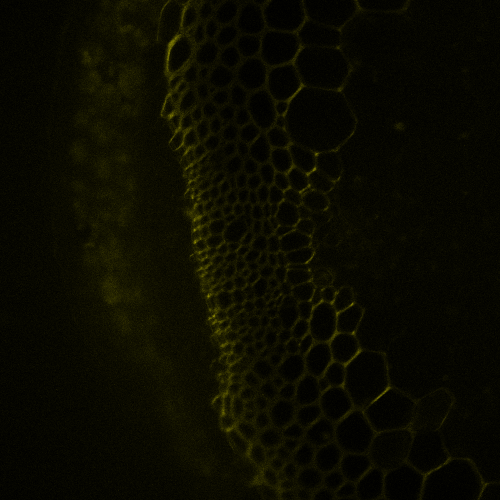

Supplement: Supplementary File 1 [file plants-03-00513-s001.zip › plants-66741-supplementary-layout/Supplementary Material-2-G-Res.gif]

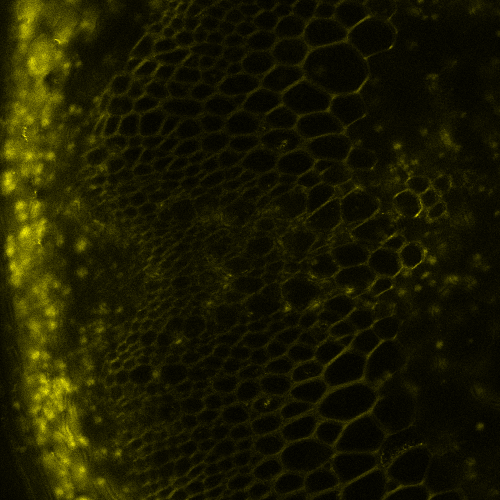

Supplement: Supplementary File 1 [file plants-03-00513-s001.zip › plants-66741-supplementary-layout/Supplementary Material-3-Cel-Res.gif]

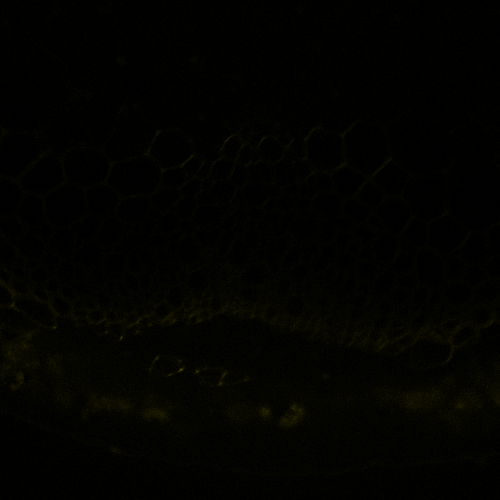

Supplement: Supplementary File 1 [file plants-03-00513-s001.zip › plants-66741-supplementary-layout/Supplementary Material-4-Gal-Res.gif]

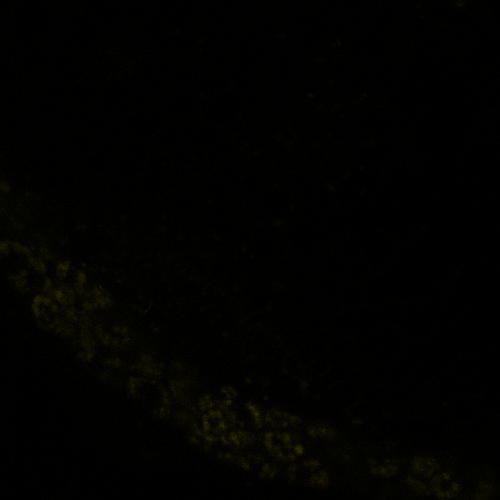

Supplement: Supplementary File 1 [file plants-03-00513-s001.zip › plants-66741-supplementary-layout/Supplementary Material-5-GG-Res.gif]

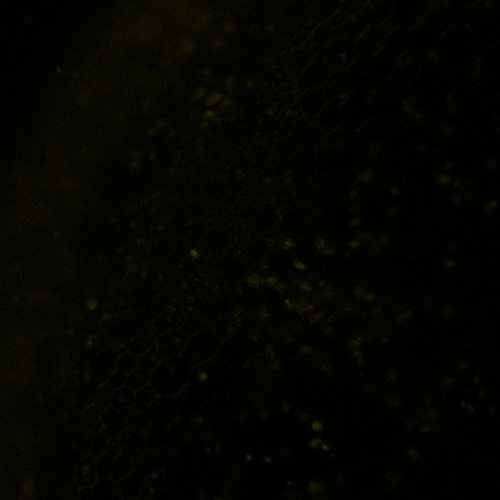

Supplement: Supplementary File 1 [file plants-03-00513-s001.zip › plants-66741-supplementary-layout/Supplementary Material-6-XG-Res.gif]
